# A practice of caution: spontaneous action potentials or artifactual spikes?

**DOI:** 10.1186/1743-0003-12-5

**Published:** 2015-01-13

**Authors:** Faezeh Jahanmiri-Nezhad, Xiaoyan Li, William Zev Rymer, Ping Zhou

**Affiliations:** Sensory Motor Performance Program, Rehabilitation Institute of Chicago; Department of Bioengineering, University of Illinois at Chicago, Chicago, IL USA; Department of Physical Medicine and Rehabilitation, University of Texas Health Science Center at Houston, TIRR Memorial Hermann Research Center, Houston, TX USA; Sensory Motor Performance Program, Rehabilitation Institute of Chicago; Departments of Physical Medicine and Rehabilitation, Physiology, and Biomedical Engineering, Northwestern University, Chicago, IL USA; Department of Physical Medicine and Rehabilitation, University of Texas Health Science Center at Houston, TIRR Memorial Hermann Research Center, 1333B Moursund St, Room 230, Houston, TX 77030 USA; Biomedical Engineering Program, University of Science and Technology of China, Hefei, China

## Abstract

**Background:**

High density surface electromyogram (EMG) techniques with electrode arrays have been used to record spontaneous muscle activity, which is important, both for supporting the diagnosis of neuromuscular diseases and for laboratory based neurophysiological investigations. This short report addresses a practical issue we have experienced during recording of spontaneous muscle activity using electrode arrays from subjects with major neuromuscular disorders.

**Findings:**

We show that recording artifacts can appear similar to spontaneous action potential spikes. Moreover, a causal filter may induce asymmetric distortions of an artifact and thus confuse it with a real action potential spike. As a consequence, for a single channel surface EMG recording, it might be difficult to judge whether a voltage transient is a real action potential or an artifact. Further investigation of the signal distributions among other channels of the array can be used to reach a more confident judgment.

**Conclusions:**

During examination of spontaneous muscle activity using electrode arrays, caution is required for differentiation of physiological signals from artifactual spikes, which is important for accurate extraction of diagnostic or investigatory information.

## Introduction

The examination of spontaneous muscle activity is an important aspect of electrodiagnostic analysis, both for supporting the diagnosis of neuromuscular diseases and for laboratory based neurophysiological investigations. An intramuscular needle electrode is routinely used for detection of spontaneous muscle activity, which can arise from the muscle fiber level disturbances (fibrillation potentials/positive sharp waves) or at the motor unit level (fasciculation potentials, multiplets, etc.). In recent years, high density surface electromyogram (EMG) techniques with electrode arrays comprised of a large number of closely spaced small recording probes or bars have achieved many applications for examination of both healthy and diseased muscles (see reviews [1–5]). One important application of such high density surface EMG recordings is to identify spontaneous muscle activity (at the motor unit level) [6–13]. This brief report presents a practical issue we have experienced during recording of spontaneous muscle activity using the electrode arrays, from subjects with neuromuscular disorders. These include amyotrophic lateral sclerosis (ALS), hemiparetic stroke and spinal cord injury. We show that recording artifacts sometimes can appear similar to spontaneous action potential spikes. Therefore, spikes in such recordings should be judged with caution as to whether they are of physiological or artefactual origins.

## Methods

We demonstrate several typical examples and discuss the discrimination of artifactual spikes from real spontaneous action potentials. A 20-channel linear electrode array (custom made, each bar width 1 mm, length 1 cm, inter-bar-distance 5 mm) and a 64-channel flexible electrode array (8 × 8 square matrix, each electrode 1.2 mm in diameter, inter-electrode-distance 4 mm for both directions, TMS International BV, the Netherlands) were used for recording spontaneous EMG activity of hand or arm muscles. The surface electrode array signals were amplified by the Refa System (TMS International), with a reference electrode located on the olecranon. Each channel also had a feedback subtraction of the mean of all the recording channels to reduce common mode noise. The collected signals (after anti-alias and SINC filters) were post-processed online with the PortiLab software (TMS International) to be filtered at 10–500 Hz with first order digital IIR filters and down sampled to 2 kHz per channel [14]. All recorded signals are in “monopolar” configuration. These signals were then converted to bipolar configuration by calculating the differential signals between two adjacent electrodes or bars along muscle fibers. This conversion was performed by offline processing. All the experimental protocols were approved by the Institutional Review Board of Northwestern University (Chicago, USA), and the tested subjects gave written consent for participating in the study.

## Findings

**Figure 1:**
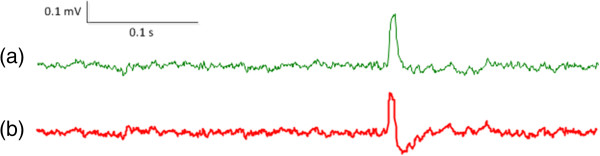
**An example of (a) an artifactual spike and (b) the distorted waveform after processing with a causal filter.**

Figure 1(a) shows an artifactual spike (the big hump located at approximately two thirds of the way into the time range) recorded by one channel of the linear electrode array. The spike was captured during recording of spontaneous EMG activity from the paretic biceps brachii muscle of a stroke subject. The linear electrode array was aligned from the proximal to distal tendon junction of the biceps brachii muscle such that innervation zone was near the middle of the array. Due to its monophasic and symmetric appearance, the spike can be recognized as an artifact. Note that it was difficult to interpret the spike as artifact solely from its large amplitude and long duration because muscle fiber reinnervation may result in the emergence of abnormally large action potentials with long durations in diseased muscles. It was further observed that if processing the signal with a causal filter (second order Butterworth high pass filter, cutoff frequency 10 Hz), the resultant waveform had a negative rebound and a gradual return to the baseline (Figure 1b). Such asymmetric distortions of the artifact may lead to the signal being confused with a real action potential spike [15]. For a single channel surface EMG recording, it remains difficult to judge whether such a spike is a real spontaneous action potential or an artifact. Further investigation of the signal distributions among other channels of the array can provide additional information to help the viewer reach a more confident judgment.

**Figure 2:**
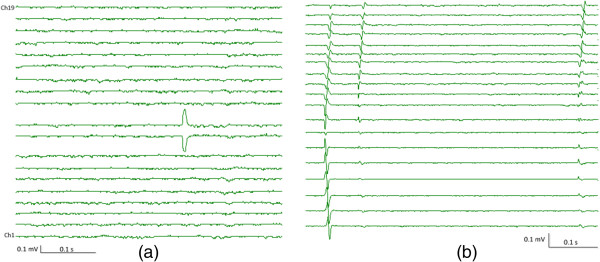
**Signal distribution among 19 channels of the linear electrode array for: (a) an artifactual spike; (b) fasciculation potentials of the biceps muscle of an ALS subject.**

Figure 2 shows such an example, where the distribution of the artifactual spike (as shown in Figure 1) among the 19 bipolar channels of the linear electrode array (placed along muscle fibers) is demonstrated in Figure 2a. We observed that the artifactual spike (actually derived from just one electrode) only occurred in 2 adjacent bipolar channels at exactly the same time, affirming its lack of physiological origin. As a comparison, Figure 2b shows three fasciculation potentials recorded from the biceps brachii muscle of an ALS subject, using the linear electrode array. The physiological origin of these potentials was confirmed by their reproducible occurrence during the long (>10 minutes) recording period. Clear action potential propagation was observed from different channels along muscle fibers, demonstrating a V-shape propagation pattern. The muscle fiber conduction velocities calculated from the inter-electrode distances and the time delays between the action potentials fell within the physiological range (3–6 m/s).

**Figure 3:**
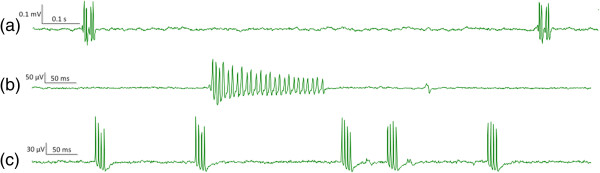
**An example of repetitive spikes from one channel of electrode arrays. (a)** Multiplets or myokymic discharges from the thenar muscle of an ALS subject; **(b)** Neuromyotonic discharges from the same muscle; **(c)** Repetitive artifactual spikes.

Figure 3 shows an example of repetitive spikes from one channel of electrode arrays, which are of physiological origin (multiplets, myokymic or neuromyotonic discharges [16, 17]) or in some instances, just recording artifacts. The signals shown in Figure 3a and b were recorded from the thenar muscle of an ALS subject using the flexible electrode array. The electrode array was placed on the muscle with columns along muscle fibers. Figure 3a shows brief bursts of single action potential spikes. The spikes within multiplets discharged multiple times in rapid succession. These bursts discharged recurrently at regular or irregular intervals up to several minutes. Figure 3(b) shows an example of neuromyotonic discharges, which have similar characteristics but bursts are prolonged and the spike amplitude may wane. In contrast, Figure 3c shows an example of repetitive artifactual spikes, which have a similar pattern to myokymic discharges. Again, for a single channel surface EMG recording, it would be difficult to judge whether such repetitive spikes are of physiological origin or just recording artifacts.

**Figure 4:**
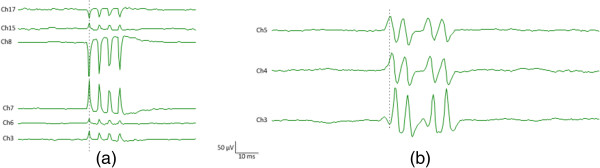
**Signal distribution among different channels of the electrode array for: (a) repetitive artifactual spikes; (b) multiplets from the thenar muscle of an ALS subject.**

Figure 4 shows the distribution of the repetitive spikes (as shown in Figure 3) on different channels of the flexible electrode array. The non-physiological origins of the repetitive artifactual spikes (Figure 4a) can be determined from two observations. First, each individual spike has monophasic and symmetric appearance; (as mentioned earlier, causal filters might change such appearance and make artifacts look more similar to real action potentials). Second, the repetitive artifactual spikes occurred at exactly the same time on different channels. In contrast, the repetitive action potential spikes demonstrate clear action potential propagation observed from different channels (Figure 4b). Indeed, we found the repetitive artifactual spikes shown in Figure 3c can be triggered when somebody called a cell phone in the proximity of the amplifier. We accidently observed such artifactual spikes when a cell phone in the proximity of the subject rang during the recording. However, such artifactual spikes are not necessarily reproducible on a different day or in a different environment.

## Discussion

To reduce artifactual spikes, it is always important to minimize possible causes of artifact. Although examination of signal distribution over an electrode array is very helpful in differentiating artifactual spikes from real action potentials, it should also be acknowledged that atypical action potential propagation patterns do not necessarily mean artifactual spikes. For example, for deeper motor units, the action potential propagation tends to be blurred by the volume-conductor properties of overlying tissue [18]. Abnormal conduction velocities in the biceps brachii muscles have also been reported in muscular dystrophy patients with the aid of a multichannel EMG system, suggesting pathological longitudinal spread of end-plates [19, 20]. In addition, the extinction of the action potential at the fiber termination may result in non-propagating waveforms which will decay more slowly than propagating action potentials and appear predominantly monophasic (particularly in pennated muscle fibers that are angled away from the skin surface) [21, 22].

Therefore, caution is required for the differentiation of spontaneous action potentials from artifactual spikes, a differentiation that is important for accurate extraction of diagnostic or investigatory information. This short report demonstrates several examples where there was a striking resemblance between spontaneous action potentials and artifactual spikes recorded during high density surface EMG examination of patients with neuromuscular disorders. We emphasize the importance of a second tier analysis (mainly with the aid of the spatial information provided by an electrode array) before accepting a spike as a spontaneous action potential.

Given the complex and potentially altered electrophysiology of neuromuscular disorders, it might be difficult to set specific rules applicable to different situations for making a definitive judgment about the nature of a given voltage transient, or spike. Nonetheless, for difficult-to-determine spikes, a comprehensive evaluation collectively using different pieces of information (such as spike distribution, amplitude, duration, shape, filter setting, electrode montage, recording environment, etc.) will routinely help reach the most reliable decision.
